# A Rare Case of Brain Metastases in an Elderly Patient With Primary Pancreatic Cancer

**DOI:** 10.7759/cureus.27578

**Published:** 2022-08-01

**Authors:** Sharad Rajpal, Hash B Taha, Lukas Kvascevicius, Sigita Burneikiene

**Affiliations:** 1 Neurosurgery, Boulder Neurosurgical Associates, Boulder, USA; 2 Integrative Biology and Physiology, University of California Los Angeles, Los Angeles, USA; 3 Medicine, Vilnius University, Vilnius, LTU; 4 Neurosurgery, Justin Parker Neurological Institute, Boulder, USA

**Keywords:** stereotactic radiosurgery, gross-tumor resection, pancreatic adenocarcinoma, brain metastasis, case report

## Abstract

Pancreatic adenocarcinoma is an extremely aggressive cancer with a low survival rate. Common sites for metastases include the liver and lungs, while brain metastases are considered extremely rare, especially in elderly patients. We present an elderly female patient who developed brain metastases 51 months after the initial diagnosis of pancreatic cancer and was treated with gross tumor resection, chemotherapy, and stereotactic radiosurgery. The treatment completely resolved her neurological symptoms but did not result in improved survival for this patient. The patient developed generalized tonic-clonic seizures, was diagnosed with leptomeningeal carcinomatosis, and died 5.5 months after tumor resection. The literature on pancreatic cancer with brain metastases is scarce, with limited guidelines for treatment strategies in this patient population. Adding this case report to the existing literature may provide additional guidance to clinicians managing patients with similar presentations.

## Introduction

Pancreatic adenocarcinoma is aggressive cancer with an overall five-year relative survival rate of 9% [1] and a 7.6 per 100,000 incidence rate occurring in North America [2]. The most common sites for metastases from metastatic pancreatic cancer stage III and above include the liver, peritoneum, lungs, and bone [3]. Although the actual incidence of brain metastases from pancreatic cancer is unknown, they are considered extremely rare (0.1%-0.6%) [4,5], with an even lower incidence of antemortem cases reported in the literature [6,7]. This number is expected to increase due to improved treatments and prolonged survival. With only 28 brain metastasis from pancreatic cancer cases occurring before death reported in the literature to date [6], we report another rare case in an elderly female patient.

## Case presentation

**Figure 1:**
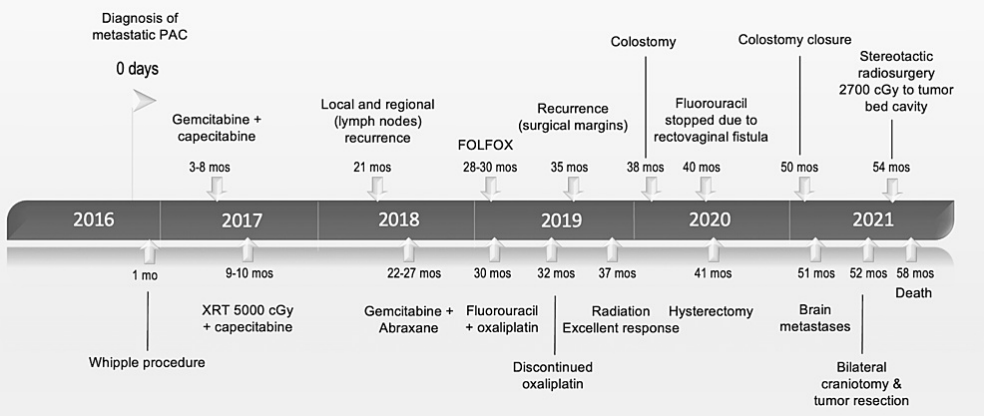
Timeline of patient’s treatment course. CGy - centi-gray; FOLFOX - chemotherapy drug consisting of leucovorin calcium, fluorouracil, and oxaliplatin; mos - months; PAC - pancreatic adenocarcinoma, XRT - radiotherapy.

**Figure 2:**
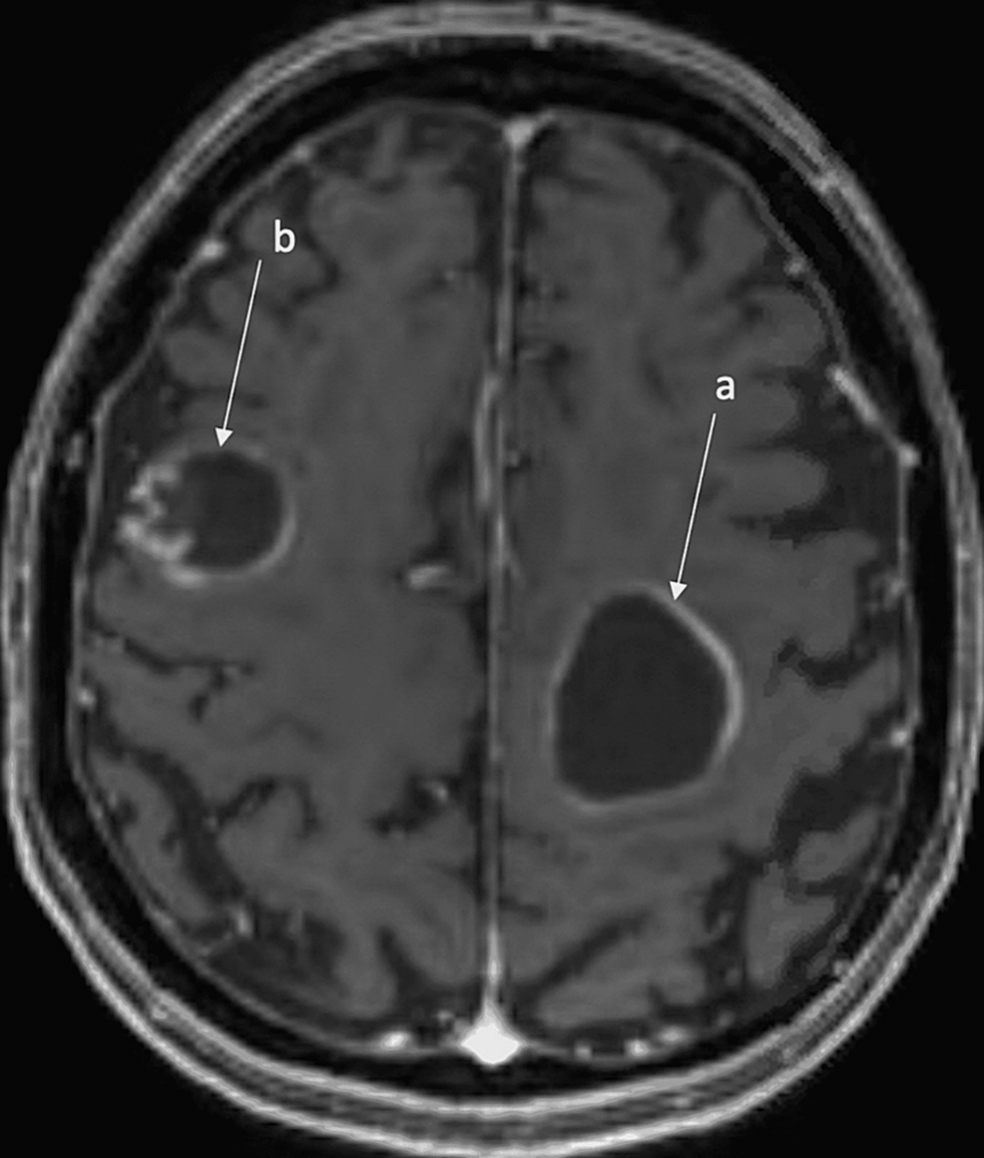
Pre-operative T1-weighted axial magnetic resonance imaging (MRI) view of the brain with contrast, preoperative scan shows (a) 31 x 30 mm cystic lesion in the frontal lobe with rim enhancement inferiorly and (b) 18 x 18 mm cystic lesion in the high frontal region with peripheral enhancement ring along the lateral border.

A very active 80-year-old female patient, Karnofsky score 80, presented to the emergency department with worsening right upper extremity (RUE) and lower extremity (RLE) weakness, difficulty with fine motor tasks, but intact comprehension and speech. Neurological exam revealed intact sensation, but weakness in the RUE and RLE: 4/5 right grip, 3/5 biceps/triceps, 1/5 right deltoid, 3/5 dorsi- and plantar flexors, and 2/5 in hip flexion. The patient had stage IV (T3, N1, M1) pancreatic adenocarcinoma with brain metastases. Seven years earlier, the patient had undergone a Whipple procedure, followed by adjuvant chemotherapy and radiotherapy (Figure 1), with excellent response and no known residual disease burden. Head computed tomography and brain magnetic resonance imaging (MRI) studies revealed right (18 x 18 mm) and left (31 x 30 mm) frontal lesions with peripheral enhancement and central necrosis with mild adjacent brain edema without midline shift, but a slight mass effect on the motor strip (Figure 2).

Operation

The patient was admitted to the hospital and underwent oncologic staging which revealed no additional primary or metastatic lesions. The patient’s case was presented to Tumor Board. The consensus of the Tumor Board was to proceed with surgery followed by adjuvant chemo- and/or radiosurgery, given the location of the tumor and the patient’s overall status. Surgery was recommended to help improve the patient’s neurologic deficit and functional status, which was not felt to improve as quickly with radiation therapy. All treatment options were reviewed with the patient, including no treatment, chemo/radiation alone, and debulking of the solitary symptomatic lesion vs. both lesions, but the patient opted for surgery on both to reduce the chance of requiring any future surgeries.

**Figure 3:**
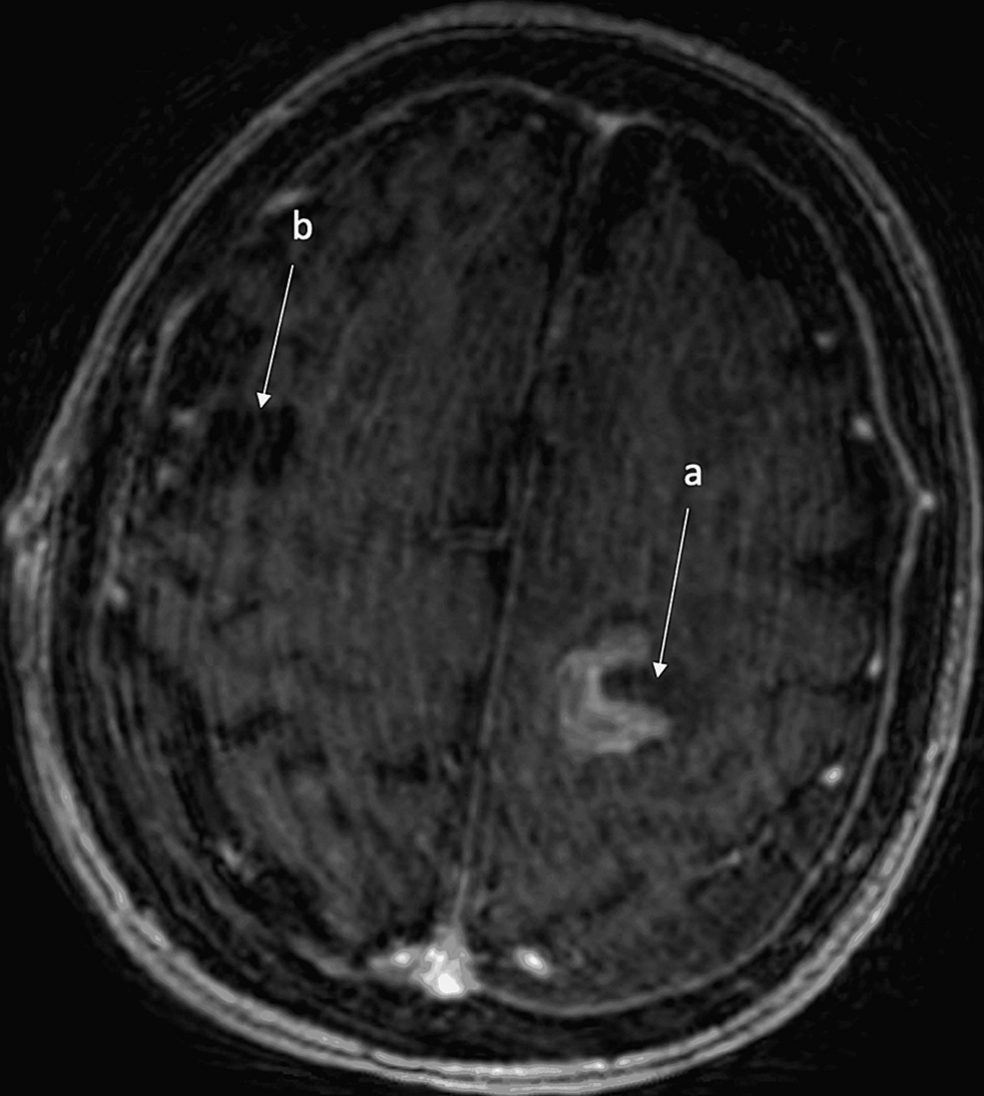
Motion degraded post-operative image demonstrates interval resection of the left frontoparietal (a) and right posterior frontal lesions (b) with a small amount of blood involving the surgical resection (left > right) without evidence of enhancement and a small left posterior convexity subdural hematoma. MRI - magnetic resonance imaging.

The patient was taken to the operating room and underwent a bifrontal craniotomy. A postoperative MRI scan (Figure 3) showed gross total resections of both lesions with deceased mass effect and without residual enhancement. Pathologic analysis of the specimens demonstrated malignant cells consistent with metastatic adenocarcinoma of pancreatic primary exhibiting moderately to well-differentiated glandular structures with associated mucin and calcification. Immunostaining was positive for cytokeratin 7, epithelial membrane antigen, CDX2 (patchy), and CK20 (focal) without expression of thyroid transcription factor - 1.

Postoperative course

Three weeks postoperatively, the patient had complete resolution of her right-sided weakness and no other neurological deficits. The patient underwent postoperative stereotactic radiosurgery (SRS) to both tumor bed cavities. The total target dose of 2,700 cGy was prescribed, 900 cGy in 3 fractions. The patient returned to the hospital 5.5 months after her tumor resection with generalized tonic-clonic seizures and severe short-term memory issues. The patient was placed on levetiracetam and a brain MRI with contrast revealed leptomeningeal enhancement concerning leptomeningeal carcinomatosis. Given the patient’s overall and rapid decline, the family and patient opted for palliative management, and she died within a few days. 

## Discussion

This case presents an elderly female patient with metastatic adenocarcinoma of pancreatic primary. Advanced age is a risk factor for developing pancreatic adenocarcinoma with cases primarily occurring in elderly patients with a median age of 70 [8]. Brain metastases from pancreatic adenocarcinoma most often occur in younger individuals with a median age of 58 and are often identified postmortem, with only 28 cases diagnosed before death (Table 1) [6]. The median time between the pancreatic adenocarcinoma diagnosis and brain metastasis development ranges between 14 and 29 months [6,9]. The longest time was reported by Lemke et al. [10]. The authors presented a 48-year-old female patient diagnosed with a right cerebellum metastasis 65 months after the diagnosis of pancreatic adenocarcinoma, which was successfully resected followed by radiotherapy with no local or distant recurrences at the 17-year follow-up. In our case, the patient’s primary cancer was successfully managed for 51 months until the development of brain metastases and leptomeningeal carcinomatosis. There are only a few reports of brain metastasis and leptomeningeal disease in patients with pancreatic cancer [11].

**Table 1:** Literature review of pancreatic adenocarcinoma patients with brain metastasis. BM - brain metastasis; BSC - best supportive care; D – days; DP - distal pancreatosplenectomy; Dx - diagnosis, LN - lymph node; M – months; MST - median survival time; N – no; Res – resection; OS - overall survival time; PD – pancreaticoduodenectomy; RT – radiotherapy; S – synchronous; Tx – therapy; Y – years; Y – yes; yo - years old. The table was obtained from Oka et al., 2021. This is an open access article distributed under the terms of the Creative Commons CC BY license.

First author	yo, sex	Operation	Metastatic site	Period between BM Dx to PDAC Dx	Treatment to BM	OS from BM Dx	Molecular Profiles
Kuratsu [12]	56yo, M 58yo, M	PD (-)	(-) Liver (S)	12 M 5 M	Ommaya + RT Res	Dead (9 M) Dead (2 W)	(-)
Chiang [13]	54yo, M	(-)	Liver (S)	S	Res + RT	Alive (> 20 M)	CK7( +), CK20(-) TTF-1(-), CDX2(-) MUC1/5AC( +), MUC2(-) KRAS^G12V^ mutation
Caricato [14]	54yo, M	PD	(-)	24 M	Res	Alive (>12 M)	(-)
Park [15]	48yo, M 51yo, M 52yo, M 62yo, M	(-)	Lung (S) Lung, Liver, Bone (S) Liver (S) Lung (S)	4 M S 5 M S	RT BSC RT BSC	MST 2.9 M (1.5M-3.8 M)	(-)
El Kamar [7]	56yo, M	(-)	Liver (S)	6 M	chemoTX	Dead (3D)	CK7( +), CK20( +) TTF-1(-)
Lemke [10]	48yo, F 66yo, M	DP DP	Liver (36 M) (-)	72 M 12 M	Res + RT Res + RT	Alive (> 10Y) Alive (> 6Y)	(-)
Matsumura [16]	64yo, M	DP	LN (12 M)	14 M	Res + RT	Alive (> 10 M)	(-)
Marepally [17]	36yo, F	(-)	Liver (S)	12 M	Res	Dead (< 1 M)	Adnab-9
Matsumoto [18]	68yo, M	(-)	Liver (S)	S	Res	Dead (3 M)	CK7( +), CK20(-)
Rajappa [19]	67yo, M	(-)	Liver (S), Lung (52 M)	48 M	Res + RT	Dead (36 M)	CK7( +), CK19( +) TTF-1(-)
Zaanan [20]	57yo, M	PD	Liver (6 M)	48 M	BSC	Dead (3D)	(-)
Rao [21]	58yo, M	(-)	Lung, Liver, Bone (S)	S	RT	Dead (<3 M)	CK7( +), CEA( +) CK20/CDX2/ TTF-1(-)
Kumar [9]	Median 61.5yo (N = 8)	PD (n = 5) DP (n = 1) Partial (n = 1) (-) (n = 1)	Lung (n = 3) Liver (n = 2) Bone (n = 3) LN (n = 3)	Median period 29 M (2 M-57 M)	Reported (n = 4): Res + RT (n = 1) Res (n = 1) RT (n = 2)	> 9Y (post Res)	(-)
Matsuo [22]	61yo, F	(-)	Ascites (S)	16 M	Res	Dead (3 W)	(-)
Sasaki [23]	72yo, F 78yo, M	(-) DP	Liver (S) Lung (5 M)	19 M 28 M	RT RT	Dead (13 M) Dead (32 D)	(-)
Oka [6]	69yo, M	DP	Lung (8 M)	8 M	Ommaya + γknife	Alive (>1 M)	CAIX( +) MUC1/5AC( +) CDX2/MUC2( +)
Our case	80yo, F	PD	Liver (S)	51 M	Res + RT	Dead (5.5 M)	CK7/EMA/CDX2/CK200 (+), TTF-1 (-)

The optimal treatment strategies for pancreatic adenocarcinoma patients with brain metastases are still debatable, but surgical resection is generally recommended, especially for cystic lesions larger than 3 cm and located in eloquent areas [24]. In the recently published Radiation Therapy for Brain Metastases Guidelines by the American Society for Radiation Oncology, SRS alone is conditionally recommended and has low-quality of evidence for patients with intact brain metastases measuring between 2 and 4 cm in diameter [25]. Given the tumor sizes, symptomatology and remission of the primary tumor, surgical resection, and adjuvant SRS was considered feasible treatment strategy for this patient. Overall, the patient did well postoperatively and recovered full strength. In hindsight, a single craniotomy to resect the symptomatic lesion would have been a less invasive strategy, but this was a very active woman who wished to return to her baseline functional status. Surgery performed seemed an appropriate course of action to address her presentation.

The median survival time was 11 months, and the 1-year local control rate was 73% in a study that evaluated tumor bed SRS after resection of brain metastases [26]. The authors noted that a lower maximum dose and SRS delay (>3 weeks) were associated with increased local recurrence. In our case, the patient survived 58 months after the initial diagnosis. Although a full resolution of her neurological symptoms was achieved for a short period of time following gross total resection and adjuvant SRS, it did not result in an overall survival benefit. According to a literature review of the 28 reported antemortem cases so far [6], the survival from diagnosis of brain metastases ranges from two weeks to more than 10 years for surgical treatment and from three days to more than one year for treatments that included best supportive care, chemotherapy and radiation therapy.

## Conclusions

We report an 80-year-old female patient treated with a gross total resection and adjuvant radiosurgery for two bilateral frontal area metastases from pancreatic cancer, which completely resolved her neurological symptoms, but did not result in improved survival for this patient. The literature on pancreatic cancer with brain metastases is scarce, with limited guidelines for treatment strategies in this patient population. Adding this case report to the existing literature may provide additional guidance to clinicians managing patients with similar presentations.

## References

[1] Siegel RL, Miller KD, Jemal A (2020). Cancer statistics. CA Cancer J Clin.

[2] Rawla P, Sunkara T, Gaduputi V (2019). Epidemiology of pancreatic cancer: global trends, etiology and risk factors. World J Oncol.

[3] Yachida S, Iacobuzio-Donahue CA (2009). The pathology and genetics of metastatic pancreatic cancer. Arch Pathol Lab Med.

[4] Oweira H, Petrausch U, Helbling D (2017). Prognostic value of site-specific metastases in pancreatic adenocarcinoma: a surveillance epidemiology and end results database analysis. World J Gastroenterol.

[5] Cannistrà M, Ruggiero M, Zullo A, Serafini S, Grande R, Nardo B (2015). Metastases of pancreatic adenocarcinoma: a systematic review of literature and a new functional concept. Int J Surg.

[6] Oka Y, Takano S, Kouchi Y (2021). Simultaneous brain and lung metastases of pancreatic ductal adenocarcinoma after curative pancreatectomy: a case report and literature review. BMC Gastroenterol.

[7] El Kamar FG, Jindal K, Grossbard ML, Mizrachi HH, Kozuch PS (2004). Pancreatic carcinoma with brain metastases: case report and literature review. Dig Liver Dis.

[8] Wang H, Liu J, Xia G, Lei S, Huang X, Huang X (2020). Survival of pancreatic cancer patients is negatively correlated with age at diagnosis: a population-based retrospective study. Sci Rep.

[9] Kumar A, Dagar M, Herman J, Iacobuzio-Donahue C, Laheru D (2015). CNS involvement in pancreatic adenocarcinoma: a report of eight cases from the Johns Hopkins Hospital and review of literature. J Gastrointest Cancer.

[10] Lemke J, Barth TF, Juchems M, Kapapa T, Henne-Bruns D, Kornmann M (2011). Long-term survival following resection of brain metastases from pancreatic cancer. Anticancer Res.

[11] O'Connor CA, Park JS, Kaley T (2021). Leptomeningeal disease in pancreas ductal adenocarcinoma: a manifestation of longevity. Pancreatology.

[12] Kuratsu J, Murakami M, Uemura S, Ushio Y (1990). Brain and skull metastases of hepatic or pancreatic cancer--report of six cases. Neurol Med Chir (Tokyo).

[13] Chiang KC, Yu CC, Chen JR (2012). Oncocytic-type intraductal papillary mucinous neoplasm (IPMN)-derived invasive oncocytic pancreatic carcinoma with brain metastasis - a case report. World J Surg Oncol.

[14] Caricato M, Borzomati D, Ausania F, Garberini A, Rabitti C, Tonini G, Coppola R (2006). Cerebellar metastasis from pancreatic adenocarcinoma. A case report. Pancreatology.

[15] Park KS, Kim M, Park SH, Lee KW (2003). Nervous system involvement by pancreatic cancer. J Neurooncol.

[16] Matsumura T, Ohzato H, Yamamoto T (2009). [A case of postoperative brain metastasis originated from pancreatic cancer which was successfully treated by resection and postoperative irradiation]. Gan To Kagaku Ryoho.

[17] Marepaily R, Micheals D, Sloan A (2009). Octreotide uptake in intracranial metastasis of pancreatic ductal adenocarcinoma origin in a patient with a prolonged clinical course. Dig Dis Sci.

[18] Matsumoto H, Yoshida Y (2015). Brain metastasis from pancreatic cancer: a case report and literature review. Asian J Neurosurg.

[19] Rajappa P, Margetis K, Wernicke G (2013). Stereotactic radiosurgery plays a critical role in enhancing long-term survival in a patient with pancreatic cancer metastatic to the brain. Anticancer Res.

[20] Zaanan A, Lequoy M, Landi B, Lievre A, Franco D, Taïeb J (2009). Brain metastases from pancreatic adenocarcinoma. BMJ Case Rep.

[21] Rao R, Sadashiv SK, Goday S, Monga D (2013). An extremely rare case of pancreatic cancer presenting with leptomeningeal carcinomatosis and synchronous intraparenchymal brain metastasis. Gastrointest Cancer Res.

[22] Matsuo S, Amano T, Kawauchi S, Nakamizo A (2019). Multiple brain metastases from pancreatic adenocarcinoma manifesting with simultaneous intratumoral hemorrhages. World Neurosurg.

[23] Sasaki T, Sato T, Nakai Y, Sasahira N, Isayama H, Koike K (2019). Brain metastasis in pancreatic cancer: two case reports. Medicine (Baltimore).

[24] Soffietti R, Abacioglu U, Baumert B (2017). Diagnosis and treatment of brain metastases from solid tumors: guidelines from the European Association of Neuro-Oncology (EANO). Neuro Oncol.

[25] Gondi V, Bauman G, Bradfield L (2022). Radiation therapy for brain metastases: an Astro clinical practice guideline. Pract Radiat Oncol.

[26] Iorio-Morin C, Masson-Côté L, Ezahr Y, Blanchard J, Ebacher A, Mathieu D (2014). Early gamma knife stereotactic radiosurgery to the tumor bed of resected brain metastasis for improved local control. J Neurosurg.

